# Evaluation of a New Sublingual Methylcobalamin Dosage Regimen for Childhood Vitamin B12 Deficiency

**DOI:** 10.3390/children12040442

**Published:** 2025-03-30

**Authors:** Sultan Aydin, Suheyla Ceren Islamoglu, Ayse Oz

**Affiliations:** 1 Department of Pediatric Hematology and Oncology, Antalya Training and Research Hospital, 07100 Antalya, Turkey; drayseoz20@gmail.com; 2 Department of Pediatrics, Antalya Training and Research Hospital, 07100 Antalya, Turkey; suheyla.islamoglu@saglik.gov.tr

**Keywords:** sublingual methylcobalamin, vitamin B12 deficiency, childhood

## Abstract

Background: Effective and timely treatment of vitamin B12 deficiency in childhood is crucial because it can lead to serious issues, including delayed growth and neuromotor development. Available treatment options include oral, intramuscular, and sublingual administration. Aim: This study investigated the efficacy of a new dosing protocol for sublingual methylcobalamin. Materials and Methods: In total, 312 patients with vitamin B12 deficiency (serum level < 250 ng/L) were divided into two groups based on their treatment type: intramuscular cyanocobalamin (Group 1) and sublingual methylcobalamin (Group 2). Group 1 included 29 (9.3%) patients, and Group 2 included 283 (90.7%) patients, with 56 (18%) patients in Group 2 undergoing treatment for childhood cancer. The sublingual methylcobalamin protocol consisted of 1 puff (500 μg) daily for children under 8 years of age and 2 puffs (1000 μg) daily for those 8 years and older, administered for 1.5 months and then three times weekly for an additional 1.5 months. Results: The mean ages in Groups 1 and 2 were 10.07 ± 6.05 years (range, 1–17 years) and 7.43 ± 5.86 years (range, 0.1–17 years), respectively. The female/male ratio was 19/10 in Group 1 and 145/138 in Group 2. The most common diagnoses were anemia (72, 22.9%), cancer (56, 18.0%), and hemangioma (40, 12.7%). The median serum levels of vitamin B12 in Group 1 were 177 ng/L before treatment, 447 ng/L after 1.5 months, and 321.5 ng/L after 3 months. In Group 2, the levels were 172 ng/L before treatment, 438 ng/L after 1.5 months, and 360 ng/L after 3 months. There were no significant between-group differences. Both groups showed a statistically significant increase in levels above 300 ng/L. Discussion: Sublingual methylcobalamin, a noninvasive treatment option, was as effective as intramuscular cyanocobalamin. This study is to compare the standard intramuscular protocol with a new dosing regimen for sublingual methylcobalamin. This study showed that it is also useful for children to hold the spray in their mouths for 1 min and avoid food intake for the next 15 min.

## 1. Introduction

Vitamin B12 deficiency is one of the most common nutritional deficiencies globally, with a prevalence ranging from 1.5% to 15.0%. Its high prevalence is attributed mainly to insufficient vitamin B12 intake, particularly in societies with lower socioeconomic status [1]. Additional causes include vegetarian diets, pernicious anemia, gastrectomy, obesity surgery, certain medications (such as proton pump inhibitors, H2 blockers, and antacids), gastric bypass, ileal resection, and, although rare, transcobalamin deficiency [2]. Vitamin B12 deficiency can lead to a range of symptoms, including weakness, loss of appetite, headache, dizziness, memory problems, irritability, fatigue, numbness in the hands and feet, depression, and learning difficulties [3,4]. In infants, it may be associated with feeding difficulties, neurodevelopmental delay, and hypotonia [3,5]. If left untreated, these conditions can become permanent. Consequently, early treatment of vitamin B12 deficiency in childhood is critical, as is managing iron deficiency resulting from irregular iron prophylaxis [6]. Treatment options include intramuscular cyanocobalamin, which is an invasive method, and oral cyanocobalamin, or sublingual methylcobalamin, which is noninvasive. Intramuscular cyanocobalamin is associated with several drawbacks, including pain at the injection site, bleeding, muscle damage, potential sciatic nerve injury, higher cost due to the need for healthcare professional administration, and hospitalization requirements. Oral cyanocobalamin is less effective for patients with malabsorption issues [7]. Sublingual methylcobalamin has recently gained importance in managing vitamin B12 deficiency because of its ease of administration and rapid absorption into the systemic circulation.

In this study, we examined whether sublingual methylcobalamin treatment, a newer approach to childhood vitamin B12 deficiency, is as effective as intramuscular cyanocobalamin. We also compared the efficacy of a new dosing regimen for sublingual methylcobalamin with the standard protocol for intramuscular cyanocobalamin.

## 2. Methods

This study involved 312 patients under 18 years of age with vitamin B12 deficiency, defined as a blood level < 250 ng/L [8], who were treated at the Department of Pediatric Hematology and Oncology at Antalya Training and Research Hospital between September 2022 and April 2023. The patients were divided into two groups based on the treatment method: intramuscular cyanocobalamin (Group 1, n = 29) and sublingual methylcobalamin (Group 2, n = 283). Of the patients receiving sublingual methylcobalamin, 56 were undergoing treatment for childhood cancer. Age, sex, vitamin B12 treatment method, and hematological parameters—including hemoglobin (Hb), red blood cell count (RBC), mean corpuscular volume (MCV), platelet count (Plt), white blood cell count (WBC), and serum vitamin B12 level—were evaluated before and after treatment through retrospective analysis.

**Intramuscular cyanocobalamin treatment group (Group 1):** Patients in Group 1 received intramuscular cyanocobalamin (Dodex^®^ Injectable; DEVA Holding A.Ş., Istanbul, Turkey) according to a structured schedule: daily administration during the first week, every other day during the second week, twice a week during the third week, once weekly during the fourth week, and once a month for the following 3 months.

**Sublingual methylcobalamin treatment group (Group 2):** Children under 8 years of age were administered one puff (500 μg) of sublingual methylcobalamin, while those aged 8 years and older received two puffs (1000 μg). Methylcobalamin (Ligone Methyl B12^®^; RC Farma, Istanbul, Turkey) was given daily for the first 1.5 months, followed by administration 3 days a week for the next 1.5 months. Patients were instructed to hold the medication in their mouths for 1 min after spraying and then swallow; they were also instructed to avoid eating or drinking for 15 min afterward. These preparations are classified as food supplements.

### 2.1. Ethics Statement

This study was conducted by the 1975 Declaration of Helsinki and was deemed ethically appropriate based on the protocol numbered 2024-191 and on the decision numbered 9/2 by the Clinical Research Ethics Committee of SBÜ Antalya Training and Research Hospital, dated 13 June 2024. Parental consent was obtained because minors were included. Data were retrospectively collected from the patients’ medical records. The patients or guardians provided written informed consent for using their medical data in this retrospective study.

### 2.2. Statistical Analysis

Data analysis was performed using IBM SPSS version 23 (IBM Corp., Armonk, NY, USA). Normality was assessed with the Shapiro–Wilk test and Kolmogorov–Smirnov test. The Mann–Whitney U test was applied to compare non-normally distributed data between binary groups, while Friedman’s test was used to compare non-normally distributed data across three or more time points. Dunn’s test was applied for multiple comparisons. Yates correction was used to compare categorical variables between groups. Quantitative data are presented as mean ± standard deviation and median (minimum–maximum), and categorical data are presented as frequency (percentage). A significance level of *p* < 0.050 was applied.

## 3. Results

In total, 312 patients were included in the analysis. Their mean age was 7.68 ± 5.92 years. Group 1 (intramuscular cyanocobalamin) included 29 (9.3%) children, while Group 2 (sublingual methylcobalamin) included 283 (90.7%) children. The female/male ratio was 19/10 in Group 1 and 145/138 in Group 2 (*p* = 0.204). The mean age was 10.07 ± 6.05 years (range, 1–17 years) in Group 1 and 7.43 ± 5.86 years (range, 0.1–17 years) in Group 2 (*p* = 0.016). The most common diagnoses were anemia (72, 22.9%), cancer (56, 18.0%), hemangioma (40, 12.7%), lymphadenopathy (33, 10.5%), neutropenia (19, 6.0%), and immune thrombocytopenic purpura, bleeding disorders, and leukopenia (18, 5.7%). Less common diagnoses included hepatosplenomegaly, thalassemia minor, polycythemia, leucocytosis, thrombocytopenia, thrombocytosis, and thrombosis. All patients diagnosed with cancer were included in Group 2.

**Figure 1:**
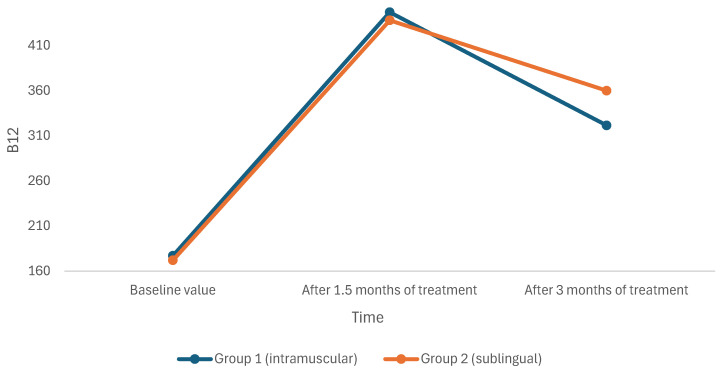
Graphical changes in serum B12 levels between two groups during the use of intramuscular and sublingual treatment.

The efficacy of intramuscular cyanocobalamin (Group 1) and sublingual methylcobalamin (Group 2) was compared in children with vitamin B12 deficiency (Figure 1). No significant difference in vitamin B12 levels was observed between the groups before treatment (*p* values in the table), at 1.5 months post-treatment, or 3 months post-treatment. In addition, the serum levels of vitamin B12 significantly increased in all patients in both groups following treatment (Table 1).

In the comparison of the complete blood count parameters between the two groups (Table 2), an increase in the serum level of Hb was noted in Group 1 at each time point (before treatment and at 1.5 and 3 months post-treatment) with intramuscular cyanocobalamin, although this change was not statistically significant. In Group 2, however, a statistically significant increase was observed over the same period with sublingual methylcobalamin treatment. Hb levels in Group 2 were lower at 1.5 and 3 months after treatment. In Group 1, there was an increase in RBC values at each time point, while Group 2 showed a statistically significant decrease in RBC at all time points. The RBC in Group 2 was lower at 1.5 months after treatment. No statistically significant differences were found in the MCV, Plt, or WBC between the two groups or over the treatment period (Table 2).

## 4. Discussion

Vitamin B12 deficiency, a public health issue affecting individuals of all ages, is particularly prevalent during periods of growth, such as infancy and adolescence, when the demand for vitamin B12 increases [8]. Early-onset vitamin B12 deficiency in newborns is often linked to insufficient maternal vitamin B12 stores [9]. Given that vitamin B12 plays a role in numerous physiological processes, its deficiency leads to ineffective erythropoiesis in the bone marrow, resulting in symptoms such as pallor, weakness, fatigue, irritability, forgetfulness, depression, cognitive decline, paresthesia, and ataxia. In infants, severe neurological effects can occur, including hypotonia, poor feeding, neurodevelopmental delay, and convulsions, secondary to impaired myelin production in the central nervous system [10]. Prompt treatment of vitamin B12 deficiency is therefore essential.

Vitamin B12 is a structurally complex molecule, typically found in the diet as deoxyadenosylcobalamin or methylcobalamin and bound to dietary proteins. It enters the stomach upon oral ingestion, where the low pH environment and pepsin degrade these proteins, releasing cobalamin. The liberated cobalamin binds with high affinity to the R protein in saliva and gastric fluid. In the duodenum, pancreatic and bile secretions break down this complex, allowing intrinsic factors produced by the stomach’s parietal cells and resistance to these enzymes to bind with the freed cobalamin. Intrinsic factors protect cobalamin from bacterial degradation and facilitate its binding to intrinsic factors, such as cobalamin receptors on the microvilli of terminal ileum mucosal cells. Once absorbed, cobalamin binds to transcobalamin 2 in enterocytes, allowing it to enter the circulation. Cyanocobalamin and hydroxocobalamin are stable forms, while deoxyadenosylcobalamin and methylcobalamin are active forms in tissues, with hydroxocobalamin being convertible to both active forms. Methylcobalamin predominates in plasma (60–80%), while deoxyadenosylcobalamin is more abundant in tissues [11,12]. These active forms function as essential coenzymes, with cyanocobalamin and hydroxocobalamin converted into methylcobalamin in the cytoplasm and deoxyadenosylcobalamin in the mitochondria [13]. Given this mechanism, methylcobalamin, the active metabolite with the highest distribution in plasma, is believed to offer a more rapid and effective therapeutic response. Furthermore, sublingual administration of methylcobalamin, which allows rapid absorption into systemic circulation through sublingual vascular structures, accelerates its efficacy.

Recent studies have examined the efficacy of sublingual methylcobalamin. Kartal and Mutlu [1] applied a standard protocol of 1000 µg sublingual methylcobalamin daily for the first 7 days, followed by administration every other day for 3 weeks. B12 levels were measured before and after treatment. The levels increased from 147.5 ± 37.7 to 602.0 ± 156.1 ng/L in the intramuscular cyanocobalamin group, from 137.2 ± 36.5 to 483.4 ± 144.8 ng/L in the sublingual cyanocobalamin group, and from 146.7 ± 40.5 to 565.5 ± 108.1 ng/L in the sublingual methylcobalamin group.

We implemented a different dosing protocol: children under 8 years of age received one puff (500 μg) of sublingual methylcobalamin, while those 8 years or older received two puffs (1000 μg) daily for 1.5 months, followed by administration three times weekly for an additional 1.5 months. This regimen was compared to the standard intramuscular cyanocobalamin regimen. B12 levels before and after treatment in the intramuscular cyanocobalamin and sublingual methylcobalamin groups were as follows: the pre-treatment levels were 177 (34–257) vs. 172 (2.55–254) ng/L, the 1.5-month post-treatment levels were 447 (268–1039) vs. 438 (81–1526) ng/L, and the 3-month post-treatment levels were 321.5 (186–608) vs. 360 (74–1500) ng/L.

A recent study compared oral cyanocobalamin, sublingual methylcobalamin, and intramuscular cyanocobalamin treatments in children aged 0 to 3 years with vitamin B12 deficiency [14,15]. The protocols included 1000 µg of oral cyanocobalamin daily for the first week, then every other day for two weeks, twice weekly for two more weeks, and weekly for 3 months. A similar frequency and dosing schedule were used for the sublingual methylcobalamin group, while the intramuscular cyanocobalamin group received 100 μg intramuscularly at the same intervals. The mean pretreatment vitamin B12 levels in the oral cyanocobalamin, intramuscular cyanocobalamin, and sublingual methylcobalamin groups were 201.1 ± 63.2 ng/L, 176.1 ± 64.2 ng/L, and 187 ± 49.2 ng/L, respectively. The post-treatment levels were 449.2 ± 285.2 ng/L, 526.1 ± 284.1 ng/L, and 427.1 ± 172.1 ng/L. Our study observed no significant differences in the Hb or Plt levels among the treatment groups.

Cyanocobalamin absorption occurs through the small intestine after binding to intrinsic factors and other cobalamin-binding proteins [16]. The pharmacokinetics of cyanocobalamin are different depending on the route of administration. Intrinsic factors are essential for oral absorption; if they are deficient or the child has pathologies that affect gastrointestinal absorption, then the effects of cyanocobalamin can be decreased. On the contrary, the formulation for sublingual methylcobalamin administration is a water-soluble compound. In conclusion, sublingual methylcobalamin has recently gained importance in managing vitamin B12 deficiency because of its ease of administration and rapid absorption into the systemic circulation.

Finally, we found that, among hematological parameters, only the Hb values in the sublingual methylcobalamin group showed a significant increase with treatment, aligning with the findings from previous studies.

## 5. Limitations

Because this was a retrospective study, one limitation was the inability to measure the patients’ methylmalonic acid and homocysteine levels. In addition, we could not assess the duration and rate of improvement in clinical symptoms, such as developmental and neurocognitive changes, with treatment. A randomized controlled trial with a larger sample size is needed to address these limitations.

## Figures and Tables

**Table 1:** Comparison of vitamin B12 levels between groups during the treatment period.

Vitamin B12 Level (ng/L)	Group 1 (Intramuscular)		Group 2 (Sublingual)		*p*
	Median (Range)	Mean ± SD	Median (Range)	Mean ± SD	
**Baseline value**	177 (34–257)	162.83 ± 56.86	172 (2.55–254)	168.10 ± 49.29	0.732 ^x^
**After 1.5 months of treatment**	447 (268–1039)	513.38 ± 198.42	438 (81–1526)	552.30 ± 324.53	0.847 ^x^
**After 3 months of treatment**	321.5 (186–608)	344.75 ± 108.39	360 (74–1500)	426.88 ± 239.87	0.136 ^x^
*p*	**<0.001** ^y^		**<0.001** ^y^		

^x^ Mann–Whitney U test, ^y^ Friedman test.

**Table 2:** Comparison of complete blood count parameters between groups.

		Group 1 (Intramuscular)		Group 2 (Sublingual)		*p*
		Median (Range)	Mean ± SD	Median (Range)	Mean ± SD	
**Hb, g/dL**	**Baseline value**	12.3 (5–17)	12.18 ± 2.22	11.7 (5.2–18.2)	11.55 ± 2.27	0.083 ^x^
	**1.5 months**	12.5 (9.6–17)	12.66 ± 1.70	11.8 (5.6–17.2)	11.76 ± 2.12	**0.038 ^x^**
	**3 months**	12.7 (10–16.8)	12.81 ± 1.50	12 (5.9–17.8)	12.15 ± 1.75	**0.037 ^x^**
*p* *		0.153		**<0.001**		
**RBC, ×10^6^/mm^3^**	**Baseline value**	4.7 (3.2–5.9)	4.72 ± 0.65	4.6 (1.2–78.6)	4.69 ± 4.49	0.101 ^x^
	**1.5 months**	4.8 (3.9–6.8)	4.87 ± 0.61	4.6 (1.8–9.7)	4.54 ± 0.92	**0.034 ^x^**
	**3 months**	4.8 (4–5.7)	4.73 ± 0.52	4.5 (2.1–6.6)	4.62 ± 0.74	0.489 ^x^
*p* *		0.442		**<0.001**		
**MCV, fL**	**Baseline value**	81.0 (52–87.5)	77.52 ± 8.63	79.7 (49.6–99)	79.07 ± 9.02	0.639 ^x^
	**1.5 months**	79.4 (56–86.8)	78.19 ± 7.00	79.5 (54.1–99.6)	78.95 ± 7.60	0.716 ^x^
	**3 months**	80.4 (55.6–86)	78.72 ± 7.03	79 (55–97)	78.96 ± 7.16	0.747 ^x^
*p* *		0.820		0.597		
**Plt, ×10^9^/L**	**Baseline value**	331 (149–513)	332.41 ± 91.42	326 (2–1372)	340.29 ± 176.37	0.945 ^x^
	**1.5 months**	323 (197–557)	325.55 ± 82.17	319 (13–1395)	326.32 ± 139.40	0.872 ^x^
	**3 months**	315 (189–391)	304.92 ± 60.58	311 (2–655)	306.15 ± 115.50	0.804 ^x^
*p* *		0.307		0.095		
**WBC, ×10^9^/L**	**Baseline value**	7.6 (3.5–27)	8.78 ± 4.66	7.9 (0.6–483)	11.76 ± 33.63	0.790 ^x^
	**1.5 months**	7.9 (3.7–15)	8.17 ± 2.45	7.8 (0.1–25)	7.88 ± 3.56	0.755 ^x^
	**3 months**	7.3 (3.3–12)	7.67 ± 2.07	7.5 (0.1–22)	7.82 ± 3.48	0.999 ^x^
*p* *		0.849		0.930		

^x^ Mann–Whitney U test, * Friedman test. Hb, hemoglobin; RBC, red blood cell count; MCV, mean corpuscular volume; Plt, platelet count; WBC, white blood cell count.

## Data Availability

The original contributions presented in this study are included in the article. Further inquiries can be directed to the corresponding author.

## References

[1] Kartal A.T., Mutlu Z.C. (2020). Comparison of sublingual and intramuscular administration of vitamin B12 for the treatment of vitamin B12 deficiency in children. Rev. Investig. Clin..

[2] Stabler S.P., Allen R.H. (2004). Vitamin B12 deficiency as a worldwide problem. Annu. Rev. Nutr..

[3] Chandra J., Dewan P., Kumar P., Mahajan A., Singh P., Dhingra B., Radhakrishnan N., Sharma R., Manglani M., Rawat A.K. (2022). Diagnosis, treatment and prevention of nutritional anemia in children: Recommendations of the Joint Committee of Pediatric Hematology-Oncology Chapter and Pediatric and Adolescent Nutrition Society of the Indian Academy of Pediatrics. Indian Pediatr..

[4] Tunçer G.O., Köker A., Köker S.A., Aba A., Kara T.T., Coban Y., Akbas Y. (2019). Infantile tremor syndrome after peroral and intramuscular vitamin B12 therapy: Two cases. Klin. Padiatr..

[5] Gupta R., Rawat A., Singh P., Gupta J., Pathak A. (2019). Infantile tremor syndrome: Current perspectives. Res. Rep. Trop. Med..

[6] Karapınar T.H., Bildik O., Köker S.A., Töret E., Oymak Y., Ay Y., Demirağ B., Vergin C. (2017). The evaluation of taking iron supplements in children aged 6 months-2 years. J. Pediatr. Res..

[7] Bensky M.J., Ayalon-Dangur I., Ayalon-Dangur R., Naamany E., Gafter-Gvili A., Koren G., Shiber S. (2019). Comparison of sublingual vs. intramuscular administration of vitamin B12 for the treatment of patients with vitamin B12 deficiency. Drug Deliv. Transl. Res..

[8] United Nations of International Children’s Emergency Fund (UNICEF) (2004). Vitamin and Mineral Deficiencies: A Global Progress Report.

[9] Rashid S., Meier V., Patrick H. (2021). Review of vitamin B12 deficiency in pregnancy: A diagnosis not to miss as veganism and vegetarianism become more prevalent. Eur. J. Haematol..

[10] Aguirre J.A., Donato M.L., Buscio M., Ceballos V., Armeno M., Aizpurúa L., Arpí L. (2019). Serious neurological compromise due to vitamin B12 deficiency in infants of vegan and vegetarian mothers. Arch. Argent. Pediatr..

[11] Parry-Strong A., Langdana F., Haeusler S., Weatherall M., Krebs J. (2016). Sublingual vitamin B12 compared to intramuscular injection in patients with type 2 diabetes treated with metformin: A randomised trial. N. Z. Med. J..

[12] Green R. (2017). Vitamin B12 deficiency from the perspective of a practicing hematologist. Blood.

[13] Devalia V., Hamilton M.S., Molloy A.M. (2014). Guidelines for the diagnosis and treatment of cobalamin and folate disorders. Br. J. Haematol..

[14] Orhan Kilic B., Kilic S., Sahin Eroğlu E., Gül E., Belen Apak F.B. (2021). Sublingual methylcobalamin treatment is as effective as intramuscular and peroral cyanocobalamin in children age 0-3 years. Hematology.

[15] Bavikar R., Kulkarni R. (2023). Sublingual Methylcobalamin in Children With Vitamin B12 Deficiency Anemia. Indian Pediatr..

[16] Scott J.M. (1997). Bioavailability of vitamin B12. Eur. J. Clin. Nutr..

